# Protective or Risk Factors for Postoperative Pancreatic Fistulas in Malignant Pathology

**DOI:** 10.3390/life11111216

**Published:** 2021-11-10

**Authors:** Alin Mihai Vasilescu, Delia Florina Andriesi Rusu, Costel Bradea, Nutu Vlad, Corina Lupascu-Ursulescu, Irene Alexandra Cianga Spiridon, Ana Maria Trofin, Eugen Tarcoveanu, Cristian Dumitru Lupascu

**Affiliations:** 1 First Surgical Clinic, “St. Spiridon” Hospital Iasi, Independentei str, no 1, 700111 Iasi, Romania; alin.vasilescu@umfiasi.ro (A.M.V.); costel.bradea@umfiasi.ro (C.B.); nutu.vlad@umfiasi.ro (N.V.); ana-maria.trofin@umfiasi.ro (A.M.T.); etarcov@yahoo.com (E.T.); cristian.lupascu@umfiasi.ro (C.D.L.); 2 Faculty of Medicine, ”Grigore T. Popa” University of Medicine and Pharmacy Iasi, 700115 Iasi, Romania; corina.ursulescu@umfiasi.ro (C.L.-U.); irene.cianga-spiridon@umfiasi.ro (I.A.C.S.)

**Keywords:** pancreatic ductal adenocarcinomas, pancreatic fistula, histological aspects

## Abstract

Introduction: Malignant tumors are associated with a low incidence of postoperative pancreatic fistulas. The presence of peritumoral fibrosis is considered the protective factor for the development of postoperative pancreatic fistulas after pancreatic resections for pancreatic ductal adenocarcinomas. Methods: We analyzed a series of 109 consecutive patients with pancreatic resections for malignant pathology: pancreatic ductal adenocarcinomas and periampullary adenocarcinomas. The incidence of postoperative pancreatic fistulas has been reported in tumor histological type, in the presence of peritumoral fibrosis, and in the association between adenocarcinomas and areas of acute pancreatitis. The data obtained were processed with the statistical analysis program SPSS, and statistically significant *p* were considered at a value <0.05. Results: For the entire study group, the incidence of postoperative pancreatic fistulas was 11.01%. The lowest incidence was observed in the group of patients with pancreatic ductal adenocarcinomas (4.06% vs. 25.72% in the group with periampullary adenocarcinoma), with a *p* = 0.002. The presence of peritumoral fibrous tissue was observed in 49.31% of cases without pancreatic fistulas, and in 54.54% of cases that developed this postoperative complication (*p* = 0.5). Also, the peritumoral fibrous tissue had a uniform distribution depending on the main diagnosis (56.14% in pancreatic ductal adenocarcinoma group vs. 37.04% in periampullary adenocarcinoma group, with a *p* = 0.08). In the group of patients who associated areas of acute pancreatitis on the resections, the incidence of postoperative pancreatic fistulas was 7.8 times higher (30% vs. 3.8%, *p* = 0.026). Conclusions: Peritumoral fibrous tissue was not a factor involved in the developing of postoperative pancreatic fistulas. The association of adenocarciomas with areas of acute pancreatitis has led to a significant increase in postoperative pancreatic fistulas, which is a significant and independent risk factor.

## 1. Introduction

Pancreaticoduodenectomy is the only curative treatment for malignant tumors located in the pancreatic head, periampullary area, or distal choledochus [1,2,3]. Although, in recent years, postoperative mortality has dropped considerably (below 5%), the rate of postoperative complications remains high [4,5,6]. The most common postoperative complications are bleeding, infectious complications, delayed gastric emptying syndrome, and pancreatic fistulas [7]. The most serious postoperative complication, with an important impact on the quality of life, is the postoperative pancreatic fistula (POPF), which is responsible for an increased risk of bleeding, abscess, sepsis, and multiple organ failure [2,8,9,10,11,12,13,14]. Patients with postoperative pancreatic fistulas have an increased risk of local tumor recurrence and a double risk of death [7,11,15,16]. The presence of postoperative pancreatic fistulas has an indirect impact on postoperative survival, and this is one of the most important causes of delayed initiation of adjuvant therapy [17].

For these reasons, the aim of our study is to identify a series of factors which can be assessed by objective methods: factors that can be used to identify the patients with a high risk for the development of postoperative pancreatic fistulas.

In 2016, ISGPS (International Study Group for Pancreatic Surgery) established the necessity for a standard definition for postoperative pancreatic fistulas, and introduced new criteria for determining severity. The major change was the replacement of grade A fistula with the term “biochemical leak”, a term that corresponds to cases of asymptomatic pancreatic leak. Grade B included the cases with invasive procedures for drainage of abdominal collections and angiographic procedures. Grade C of postoperative pancreatic fistulas included the patients with organ failure, with surgical reinterventions, or death [18].

## 2. Methods

Our study is a retrospective one, which included a series of 109 consecutive patients with pancreatic resections for malignant pancreatic and periampullary tumors. Selection criteria included patients with pancreatic resections (pancreatoduodenectomy, distal pancreatectomy, pancreatic biopsy) for pancreatic ductal adenocarcinomas and periampulary adenocarcinomas, histologically confirmed. Patients with malignant neuroendocrine tumors, mucinous carcinomas, or other carcinomas other than adenocarcinomas were excluded from the study. All patients included in the study had signed, at the time of hospitalization, the consent for publication and the surgical procedure, and an agreement to participate in clinical trials and research. We analyzed the incidence of postoperative pancreatic fistulas, its association with peritumoral fibrous tissue and areas of acute pancreatitis, and the distribution of these histological aspects, depending on the diagnosis.

Resected tumors were fixed and embedded in paraffin to obtain tissue preservation in a reproducible manner. The paraffin met the conditions for a good preservation of morphologic details after embedding in a support. Each tissue fragment was initially fixed in formalin, then processed according to the standard protocol for histological examination. The sectioning of the blocks with tissues embedded in paraffin were made with the microtome, obtaining sections of 4–5 µm. The sections thus obtained were fixed on the slides, and subsequently subjected to hematoxylin eosin staining. Hematoxylin-eosin-stained slides of the resection specimens were reviewed by a pathologist specialist, who was blinded to all clinical data, and the diagnosis was reconfirmed by a histopathology phisician. Microscopically, in cases where pancreatic ductal adenocarcinoma was present, glandular tissue composed of: malignant epithelial cells, with low mitotic activity; and mucin-producing column cells with eosinophilic cytoplasm, with large and ovoids nuclei and nucleoli (which are not normally found in cells ducts). Microscopically, fibrous tissue contains an extracellular protein matrix, which contains hyaluronic acid, collagen, fibroblasts, stellate pancreatic cells, and inflammatory cells. Periampullary adenocarcinomas intestinal type are tubular carcinomas, and, microscopically, have cribriform glands, columnar cells, and elongated and oval nuclei which are located at the base.

Patients’ clinical data were obtained from observation sheets. Information was selected, such as age, gender, comorbidities, symptomatology, laboratory analyzes followed in dynamics, imaging explorations, operating protocol, and postoperative evolution.

The diagnosis of pancreatic fistula was established in patients who had three times the value of amylases in the fluid in the drain tube compared to the normal value, or a persistence of drainage. The quantification of the drainage persistence was both in terms of quantity (drainage measured in milliliters) and in terms of duration (measured in days), and by information found in the observation sheets, and the patient’s evolution.

Microscopic examination of pancreatic resections pieces revealed the association of areas of acute pancreatitis with adenocarcinomas. In these cases, patients did not show preoperative symptoms specific to acute pancreatitis or increases in preoperative values of amylases and lipases.

## 3. Statistical Analysis

To perform statistical analysis, the SPSS v.24 was used, with statistical significance established at *p* < 0.05. Continuous variables were reported as mean with standard deviation. Comparisons between the analyzed groups were performed using the t-Student test, ANOVA, Kruskal–Wallis, or Mann–Whitney U Test for continuous variables. The homogeneity of the series was verified regarding the statistical differences between the variances of the series by the Levene test (Levene Test of Homogeneity of Variances). The correlations between certain parameters were tested using the Pearson test, by evaluating the correlation coefficient r. Qualitative variables were presented as absolute (*n*) and relative (%) frequencies, and comparisons between groups were made based on the results of non-parametric M-L, Yates, or PearsonChi-square tests. Univariate and multivariate analysis of prognostic factors for complications was performed using the Logistic regression model. The power of univariate prediction of risk factors was assessed using the ROC curve based on the value of the area under the curve (AreaUnder the Curve: AUC).

## 4. Results

**Figure 1:**
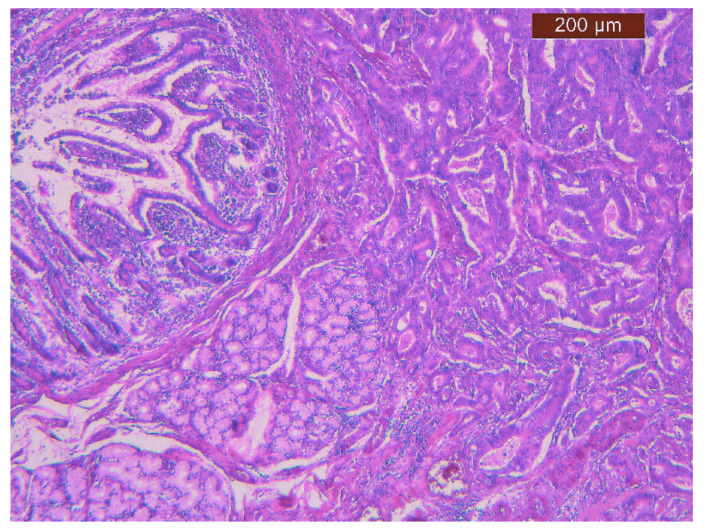
Hematoxylin eozine staining x5—microscopic appearance of periampullary adenocarcinoma.

**Figure 2:**
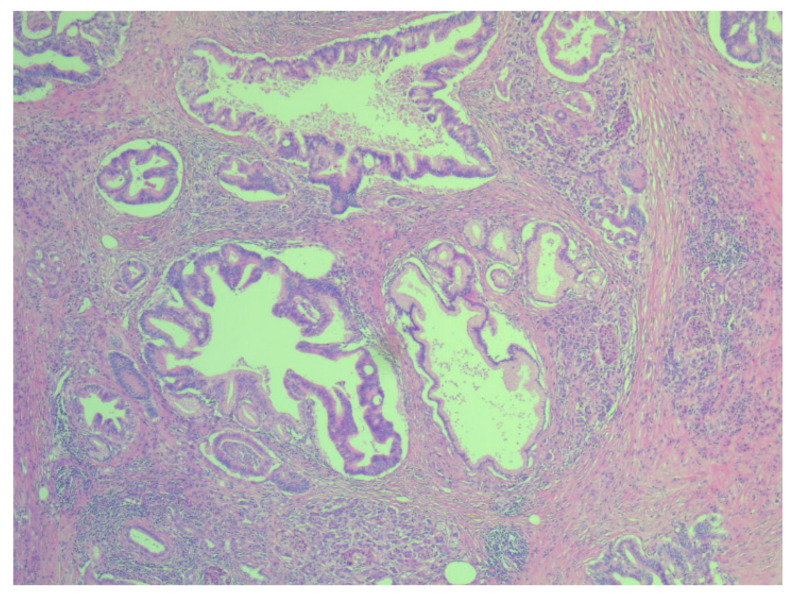
Hematoxylin eosin staining x4—microscopic appearance of pancreatic ductal adenocarcinoma.

Our study group included 109 patients (55 males and 54 females) aged between 37 and 79 years. The median age of patients with pancreatic ductal adenocarcinoma was 60.5 ± 10.52, and that of patients with periampullary adenocarcinomas was 59.49 ± 9.04. According to the histological diagnosis, 35 patients had periampullary adenocarcinomas, and 74 pancreatic ductal adenocarcinomas (Figure 1 and Figure 2).

Among the types of surgeries, pancreatoduodenectomies predominated (*n* = 89), followed by pancreatic biopsies (*n* = 12) and distal pancreatectomies (*n* = 8).

Both the group of patients with pancreatic ductal adenocarcinoma and periampullary adenocarcinomas had a series of associated comorbidities, but without a significant difference in their distribution (Table 1). Hypertension predominated in both groups of patients. The incidence of diabetes was almost three times higher in the group of patients with pancreatic ductal adenocarcinoma, and the incidence of obesity was almost double in the group of patients with periampullary adenocarcinomas.

The incidence of pancreatic fistulas on the entire study group was 11.01%. Most patients had grade B POPF (*n* = 8), one patient had grade A POPF after a pancreatic biopsy, and two patients had grade C POPF. For the two patients with grade C POPF, surgical reoperation was required, with the restoration of the pancreato-jejunal anastomosis.

No significant association was found between postoperative pancreatic fistulas and the gender (*p* = 0.606) or age (*p* = 0.605) of the patients.

No statistically significant association was found on our study group between the risk of postoperative pancreatic fistula production and the type of surgical resection (*p* = 0.537). The occurrence of postoperative pancreatic fistulas predominated in the group of patients with pancreatoduodenectomy, where the incidence was 12.4%. In this group, the operating team preferred pancreato-jejunal anastomosis “duct to mucosae”. In only three cases, a pancreato-gastric anastomosis was performed. In the group of patients with distal pancreatic resections, closure of the pancreatic trance was performed by manual suturing.

The lowest incidence of pancreatic fistulas was observed in the group of patients with pancreatic ductal adenocarcinomas (4.06% vs. 25.72% in the periampullary adenocarcinomas, with *p* = 0.002).

**Figure 3:**
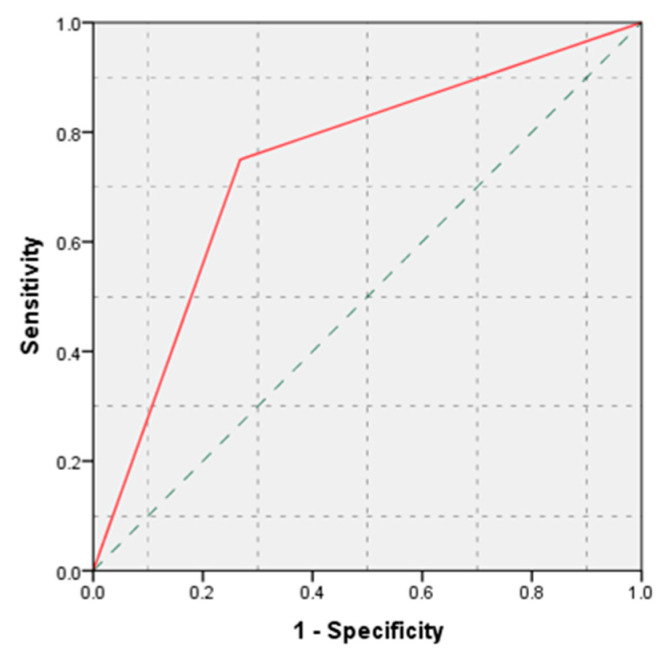
ROC curve: histological diagnosis versus pancreatic fistula.

According to statistical analysis, histological diagnosis demonstrated an increased accuracy in predicting the occurrence of postoperative pancreatic fistulas (AUC = 0.741, *p* = 0.007, 95% CI: AUC → 0.590–0.892) (Figure 3).

**Figure 4:**
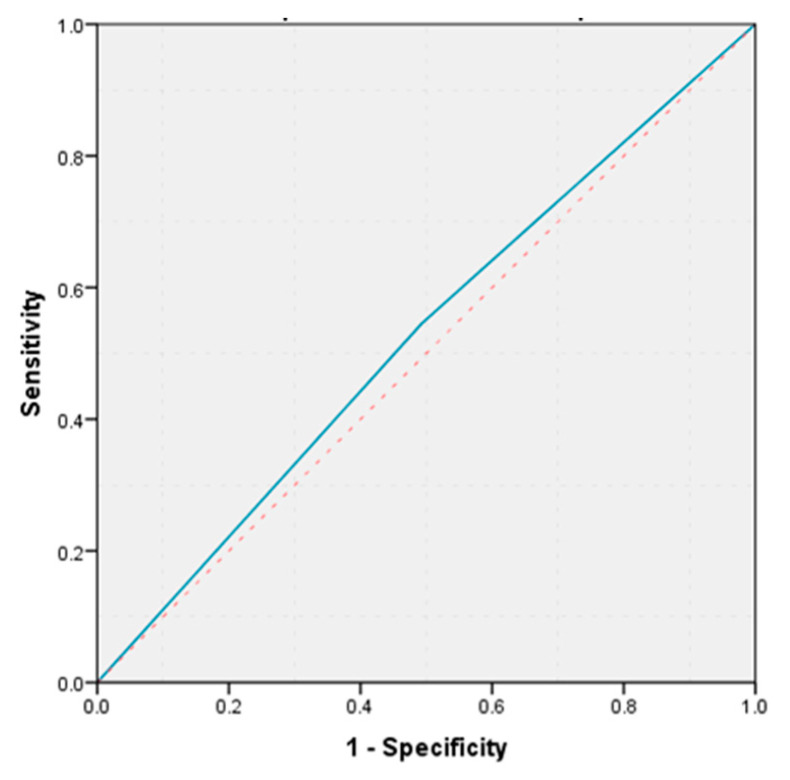
ROC curve peritumoral fibrous tissue vs. pancreatic fistula.

The presence of peritumoral fibrous tissue was objectified in 56.14% of patients with pancreatic ductal adenocarcinomas, and 37.04% in those with periampullary adenocarcinomas, without a statistically significant difference in fibrous tissue distribution according to histological diagnosis (*p* = 0.08). The group of patients with peritumoral fibrosis included patients who underwent microscopic examination of tumors delimited by desmoplastic stroma and fibrous tissue. The incidence of pancreatic fistulas was 11.9% in the group of patients without peritumoral fibrosis, and 14.3% in the group with peritumoral fibrosis, without a statistically significant difference in the two groups (*p* = 0.5). The calculated value of the area under the ROC curve demonstrates that the presence of peritumoral fibrous tissue has a weak predictive power on the occurrence of postoperative pancreatic fistula ROC (AUC = 0.526, *p* = 0.781, 95% CI: AUC → 0.343–0.710) (Figure 4).

**Figure 5:**
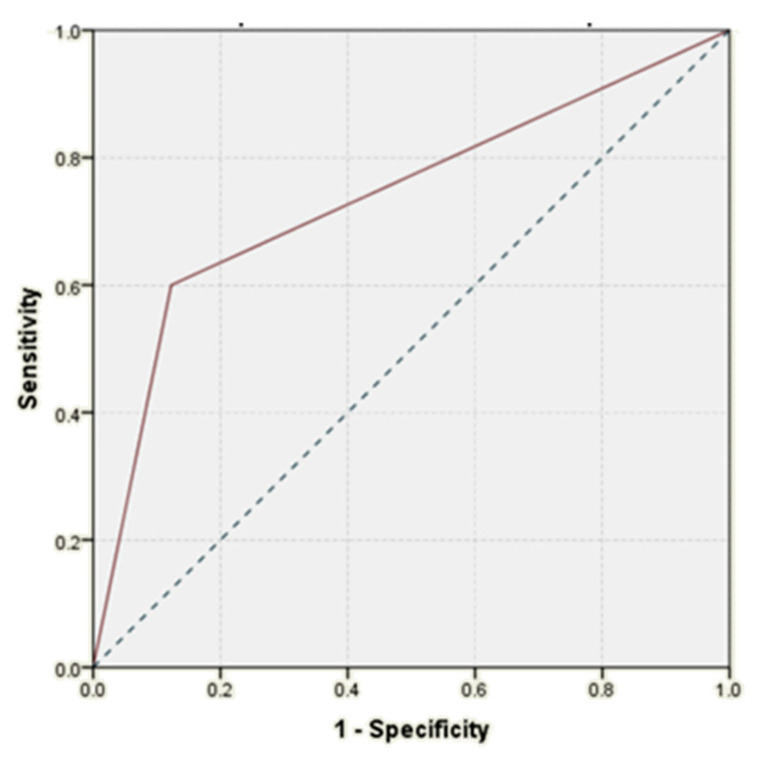
ROC curve: areas of acute pancreatitis associated with adenocarcinomas vs. pancreatic fistula.

The coexistence of acute pancreatitis areas with adenocarcinomas on the resection pieces was associated with a 7.8-fold increase in the incidence of postoperative pancreatic fistulas (30% vs. 3.8%, with a *p* = 0.026). Areas of acute pancreatitis, associated with adenocarcinomas, demonstrated a good accuracy in predicting the risk of occurrence of pancreatic fistulas (AUC = 0.739, *p* = 0.079, 95% CI: AUC → 0.474–0.998) (Figure 5).

Multivariable analysis showed that the histological diagnosis and the areas of acute pancreatitis associated with adenocarcinomas were independently associated with postoperative pancreatic fistulas (Table 2).

## 5. Discussions

The risk factors involved in the production of postoperative pancreatic fistulas can be grouped into modifiable and unchangeable risk factors. In order to eliminate the biases related to the surgeon’s experience, the subjective interpretation of the pancreas texture, the surgical technique adopted, or the possible intraoperative complications that may occur, in the current study, we turned our attention to unchangeable risk factors.

Studies in the literature have shown that pancreatic resections for malignant tumors have been associated with a reduced incidence of postoperative pancreatic fistulas [8,19,20]. The incidence of postoperative pancreatic fistulas in traumatic pancreatic surgery can reach up to 38%, according to a study conducted on an impressive group of patients in a trauma center [21].

Our study analyzed the incidence of pancreatic fistulas according to the histological type of malignant tumors, thus highlighting that not only the character of malignancy is relevant, but so too is the starting point of the primary tumor. Thus, it was observed that there was a significant difference between the presence of pancreatic fistulas in the group of patients with periampullary adenocarcinomas, and a 6.3-fold lower incidence in the group of patients with pancreatic ductal adenocarcinomas.

The hard pancreatic structure due to the peritumoral fibrous tissue is thought to reduce the risk of pancreatic fistulas for patients with pancreatic ductal adenocarcinomas [22], but this was not confirmed in our study group, where there were no statistically significant differences in distribution of peritumoral fibrous tissue depending on histological diagnosis.

Fibrous tissue contributes to changes in the gland texture of the pancreas, an aspect associated with reducing the risk of postoperative pancreatic fistulas. A soft structure of the pancreas can increase the risk of pancreatic fistula up to 10 times compared to a hard gland [23]. A series of factors can be responsible for leading to a decrease in the incidence of pancreatic fistulas in patients with a hard structure of the pancreas: (1) the prolonged pancreatic duct obstruction, which leads to a dysfunction of exocrine pancreatic function; (2) pancreatic fibrosis; (3) low risk of injury of pancreatic tissue [2]. Patients with a soft pancreas present an insecure suturing and knotting of the pancreatico-jejunal anastomosis, and a higher risk of the damage to the pancreatic tissue [2].

A multicentre study of the French Surgical Association confirmed that the rate of pancreatic fistulas was significantly higher in patients with normal or soft pancreatric parenchyma than in hard or fibrous pancreatic tissue. Also, the study concluded that the pancreatic texture can predict the occurence of pancreatic fistulas in patients with adenocarcinomas [20].

In his study, Callary observed that the soft gland texture and narrowed pancreatic duct diameter demonstrate that exocrine function is generally preserved, which is more susceptible to ischemia and injury [24].

The gland structure is a subjective factor that depends on the surgeon’s experience, and there is not a unitary standard [23], but our study eliminated this bias by microscopic confirmation of peritumoral fibrous tissue that contributes to a hard texture.

The results of our study showed that the occurrence of postoperative pancreatic fistulas did not depend on the structure of the pancreas, and other factors are involved in this process.

Martin et al. also concluded in their study that the pancreatic texture and the duct size were not associated with the development of pancreatic leak after distal pancreatectomy [25].

The process involved in the development of postoperative pancreatic fistulas is a complex one, which includes a multitude of factors that can interact [8]. One of the most important factors involved in this process is the exocrine hypersecretion of the pancreas, which plays a decisive role in producing this postoperative complication [23]. Pancreatic exocrine hypersecretion is found in acute pancreatitis, triggering and maintaining pancreatic fistulas.

To our knowledge, there are no studies that have found correlations between the association between adenocarcinomas and areas of acute pancreatitis with an increased risk of postoperative pancreatic fistula. The results of our study showed that when malignant tumors were associated with areas of acute pancreatitis, the incidence of pancreatic fistulas had a significant increase of 7.8 times higher. Cases with histologically confirmed areas of acute pancreatitis had no specific clinical symptoms. Histological confirmation of areas of acute pancreatitis being associated with adenocarcinomas is an important predictive factor, and can be considered one of the most important independent risk factors involved in the occurrence of postoperative pancreatic fistulas.

A study published in 2015 looked at the long-term effects of the association of pancreatic cancer with acute pancreatitis, but this study included patients with clinically relevant, moderate, and severe acute pancreatitis. Thus, Feng et al. found that pacreatic cancer associated with clinically relevant acute pancreatitis had a poor survival time and early reccurence within 12 months [26].

Some studies show that up to 13.8% of cases of pancreatic cancer may be associated with acute pancreatitis [26,27,28]. Feng affirms that the onset of acute pancreatitis may be due to cancer progression itself or to complications of the diagnostic and therapeutic interventional procedures used in pancreatic carcinoma treatment, such as endoscopic retrograde cholangiopancreatography, surgery, and chemotherapy [26]. Acute pancreatitis is one of the most common complications after retrograde cholangiopancreatography, and one-third of these patients develop moderate or severe forms of acute pancreatitis [26,29].

Risk factors for pancreatic fistulas differ depending on the type of pancreatic resection. In the case of distal pancreatic resections, emphasis is placed on how to close the pancreatic trance of the remaining pancreas. The way to reduce the appearance of fistulas is to close the distal pancreas using a stapler [4,10]. Clinical trials have not demonstrated the superiority of the use of a hemostatic adhesive in the distal pancreatic trance [10]. Jiwani et al. affirm that the risk of pancreatic fistula increases considerably when distal pancreatic resections also involve multiorgan resections [13]. On the contrary, Xia et al. consider that extended lymphadenectomy increases the incidence of pancreatic fistulas after distal pancreatic resections [30].

For pancreatoduodenectomies, a major role in the development of postoperative pancreatic fistulas is the type of pancreato-digestive anastomosis. Pancreato-gastric anastomosis offers a series of advantages that reduce the risk of fistula, such as: (1) the location of the stomach in the proximity of the remaining pancreas; (2) rich vascularization of the stomach; and (3) gastric acid environment [10]. Gastric acid environment has the role of inhibiting the activation of pancreatic enzymes, the rich vascularization of the stomach reduces the risk of ischemia in the pancreato-gastric anastomosis, and the anatomical location of the stomach and pancreas provides a decrease in tension of the anastomosis [10]. However, the use of pancreato-gastric anastomosis has a major disadvantage: it is associated with an increase in postoperative bleeding [10,14]. In a clinical study that observed the long-term effects of pancreato-digestive anastomosis, Benini et al. found that exocrine pancreatic function was much more affected after pancreato-gastric anastomosis, and was also associated with a decrease in vitamin D levels, and with malabsorption of fats [31].

For these reasons, pancreato-jejunal anastomosis was preferred in our surgery department, and in most cases, it was “duct to mucosae”.

A controversial topic is the use of internal stents versus no stents for pancreato-jejunal anastomosis, but the majority of reviews did not report any differences in fistula rate, mortality, or morbidity [14].

## 6. Conclusions

The postoperative pancreatic fistula remains an important problem for postoperative management of patients, with pancreatic malignant tumors being responsible for an increased risk of postoperative morbidity and mortality. Peritumoral fibrous tissue is not a factor involved in the developing of postoperative pancreatic fistulas. The association of adenocarciomas with areas of acute pancreatitis has led to a significant increase in postoperative pancreatic fistulas, which is a significant and independent risk factor. Factors such as age, gender, and type of pancreatic resection had no role in the development of pancreatic fistulas.

## Figures and Tables

**Table 1:** The presence of comorbidities depending on the histological diagnosis.

Comorbidities	Diagnostic		*p*-Value
	Pancreatic Ductal Adenocarcinoma (Absent/Present) (*n* = 74)	Periampullary Adenocarcinoma (Absent/Present) (*n* = 35)	(95% CI)
Obesity	64/10	27/8	0.224
	86.5%/13.5%	77.1%/22.9%	
Diabetes	57/17	32/3	0.066
	76.7%/23.3%	91.4%/8.6%	
Hypertension	52/19	20/9	0.702
	73.2%/26.8%	69%/31%	
Other cardiac pathologies	55/16	21/8	0.625
	78.6%/21.4%	72.4%/27.6%	

**Table 2:** Multivariable logistic regression model results for the association with pancreatic fistula.

Pancreatic Fistula	OR (Odds Ratio)	OR 95% CI Min	OR 95% CI Max	*p*-Value
Sex	0.248	0.033	1.88	0.178
Histological diagnosis	12.84	1.29	127.29	0.029
Peritumoral fibrosis	4.68	0.605	36.32	0.139
Adenocarcinomas associated with acute pancreatitits	11.34	1.34	95.66	0.026

## Data Availability

The data published in this research are available on request from the first and last author and corresponding author.
